# Establishment of a nine‐gene prognostic model for predicting overall survival of patients with endometrial carcinoma

**DOI:** 10.1002/cam4.1498

**Published:** 2018-04-17

**Authors:** Jianchao Ying, Qian Wang, Teng Xu, Jianxin Lyu

**Affiliations:** ^1^ Key Laboratory of Laboratory Medicine Ministry of Education Zhejiang Provincial Key Laboratory of Medical Genetics School of Laboratory Medicine and Life Science Wenzhou Medical University Wenzhou China; ^2^ Department of Clinical Laboratory Wenzhou People's Hospital The Third Clinical Institute Affiliated to Wenzhou Medical University Wenzhou China

**Keywords:** Endometrial carcinoma, genomewide expression profiles, overall survival, prognostic model, signature gene

## Abstract

Endometrial carcinoma (EC) is the most common malignant tumor of the female genital tract in developed countries. The prognosis of early stage EC is favorable, but a subset faces high risk of cancer progression or recurrence. EC has a poor prognosis upon progression to advanced or metastatic stages. Therefore, our goal is to build a robust prognostic model for predicting overall survival (OS) in EC patients. In this study, 1571 genes were identified as being associated with OS based on genomewide expression profiles using a training dataset. Kyoto Encyclopedia of Genes and Genomes enrichment analysis revealed that these genes were involved in various cancer‐related signaling pathways. Nine signature genes were further selected using stepwise selection, and their potential role in the development of EC was demonstrated by performing differential expression analysis between EC and normal uterine tissues. A prognostic model that aggregated these nine signature genes was ultimately established and effectively divided EC patients into two risk groups. OS for patients in the high‐risk group was significantly poorer compared with that of the low‐risk group. This nine‐gene model was subsequently validated and evaluated using the TCGA dataset and shown to have a high discriminating power to distinguish EC patients with an elevated risk of mortality based on the FIGO staging system and other prognostic factors. This study provides a novel prognostic model for the identification of EC patients with elevated risk of mortality and will help to improve our understanding of the underlying mechanisms involved in prognostic EC factors.

## Introduction

Endometrial carcinoma (EC), a cancer arising from the endometrium, is the most common gynecological tumor in developed countries 1, and its prevalence is increasing. The five‐year survival rate of EC following appropriate treatment ranges from 74% to 91% 2. To stratify patients into distinct prognostic groups, EC and other cancers of the female gynecologic system are most commonly staged using guidelines provided by the International Federation of Gynecology and Obstetrics (FIGO) based on findings at either clinical examination and/or surgical exploration 3. The FIGO staging system is an overriding prognostic factor for EC with survival declining as stage at diagnosis increases 4.

Endometrial carcinoma is often diagnosed at an early stage due to relatively frequent vaginal bleeding as a first symptom 5. Nevertheless, a subset of early stage EC may exhibit a high risk of cancer progression or recurrence, and EC has poor prognosis in response to conventional chemotherapy upon progression to advanced or metastatic stages 6. Therefore, the need to identify predictive biomarkers that help clinicians to guide rational therapy, for example determine early stage patients who might benefit from more aggressive therapy, is urgent 7. Histological type, according to traditional microscopic parameters, has consistently been proved to be an important predictor of survival, but also a determinant for the extent of the initial surgical procedure and subsequent use of adjuvant therapy 8. Histological typing correlates not only with prognosis, but also with the molecular alterations, expression and methylation profiles of each tumor type 9, 10. In addition to FIGO stage and histological type, other prognostic factors contributing to survival include histological grade, age at diagnosis, and tumor size 11.

However, given the limitations of FIGO staging system and histological classification for prognostic prediction 12, incorporation of molecular and genetic characteristics into classification systems may provide more valuable information for prognosis and predicting response to novel therapies 13. At present, significant efforts have sought to identify molecular markers, and gene expression profiling has been verified as a promising tool to classify tumors and predict cancer prognosis 14. A series of novel candidate prognostic markers 15, 16, 17, 18 have been discovered and confirmed to potentially improve the diagnosis and prognosis of EC. Prognostic models 7, 12, 19, 20 that aggregate several signature genes/proteins based on gene expression profiles or protein arrays also have been constructed, but these models are only effective for partial stages and/or grades of EC. Moreover, the developed prognostic signatures are difficult to apply widely. Therefore, a prognostic model with high discriminating power to effectively assist prognosis prediction for each stage or type is required in clinical practice.

Given that overall survival (OS) is traditionally regarded as the ultimate measure of treatment benefits, OS was used as the endpoint to develop or evaluate the prognostic model in this study. We first sought to identify genes associated with OS based on genomewide expression profiles of EC patients. We further selected optimal signature genes to construct a robust prognostic model. The prognostic model was subsequently validated and evaluated using the TCGA dataset, indicating its potential prognostic value for EC patients.

## Materials and Methods

### EC datasets and data processing

The EC dataset (*N* = 521 for primary EC tumors, marked as “TCGA dataset” in Table S1) was limited to RNA‐Seq data (reads counting with HTSeq), and its corresponding clinical information was download from the TCGA database (The Cancer Genome Atlas, http://cancergenome.nih.gov/). Among 521 EC samples, eighty EC samples were chosen randomly as the training dataset, and the remaining 441 samples were used as validation dataset (marked in Table S1). “DESeq2” function implemented in BRB‐ArrayTools (http://linus.nci.nih.gov/BRB-ArrayTools.html) was employed to transform and normalize the count data. To solve the imbalance between the tumor and normal data, which can cause inefficiency in differential expression analyses, the expression dataset (*N* = 181 for primary EC tumors from TCGA and *N* = 78 for normal uterus tissues from GTEx) was download from the UCSC Xena project (http://xena.ucsc.edu/) that recomputed all raw RNA‐Seq data based on a standard pipeline to minimize differences from distinct sources.

### Identification and functional enrichment analysis of genes associated with OS

OS‐related genes were identified by performing univariate Cox regression using BRB‐ArrayTools. Enrichment analyses of Kyoto Encyclopedia of Genes and Genomes (KEGG) pathways were conducted using clusterProfiler package 21 in R. The hypergeometric test statistical method was applied, and whole human genes were used as background genes. Only pathways with a *P*‐value threshold of <0.05 were shown and considered as significant enriched functional categories. To further narrow down the gene size, a stepwise selection method implemented in survival package was employed to select optimal signature genes. Using the scipy package in Python, the Mann–Whitney *U* test was performed to examine the differential expression of signature genes between EC and normal uterine tissues.

### Unsupervised hierarchical clustering and Kaplan–Meier survival analysis

Unsupervised hierarchical clustering was performed using “heatplot” function in R, which used an average agglomeration method with the correlation distance. Kaplan–Meier curves for two diverse groups were plotted using the “survfit” function in survival package. Hazard ratios (HR) and *P*‐values from log‐rank tests were calculated using the “survdiff” function.

### Establishment and evaluation of the prognostic model

The survival risk prediction tool implemented in BRB‐ArrayTools was used to compute the regression coefficient for each gene in the training dataset. The survival risk score is calculated by summing the product of the expression level of a gene and its corresponding regression coefficient. The leave‐one‐out cross‐validation (LOOCV) method was employed to evaluate the accuracy of the score system. Training patients were partitioned into two risk (high‐ and low‐risk) groups according to the 50th prognostic index percentile. Then, Kaplan–Meier curve analysis was performed. For validation of this model in TCGA dataset, the risk score for each patient was calculated using the coefficient obtained from the training dataset. The prognostic indexes (used for plotting ROC curve in Fig. S1C) using the panel of nine‐gene signature (*PDLIM1*,* FBP1*,* NLRC3*,* ST6GALNAC1*,* C4BPA*,* PPP2R3A*,* TRIM46*,* EPH2,* and *PRRG1*) proposed by O'Mara et al. 20 were calculated for each patient as previously described. The receiver operating characteristic (ROC) curve was plotted for 5‐year OS prediction to estimate the sensitivity and specificity of the prognostic model. The optimal cutoff risk score was obtained based on the maximum Youden index in the ROC curve and was used to divide EC patients into high‐ and low‐risk groups. The Kaplan–Meier curves for these two diverse groups were plotted using “survfit” function. Using “survdiff” function, HR and *P*‐values were calculated to evaluate the discrimination of this prognostic model. The molecular classification for EC patients was obtained from the TCGA study 10. In addition, the chi‐square test was used to analyze the association between this prognostic model and the molecular classification of EC.

## Results

### Identification of OS‐related genes in EC based on genomewide expression profiles

In this study, eighty EC samples with corresponding observed (survival or censoring) time and censoring status were selected as the training dataset. RNA‐Seq data of these samples, which included expression values of 16,560 genes, were transformed and normalized using BRB‐ArrayTools. Genes associated with OS were identified by performing univariate survival analysis using Cox proportional hazard regression model with a *P*‐value threshold of 0.05. As a result, a total of 1571 OS‐related genes were obtained (Table S2).

**Figure 1:**
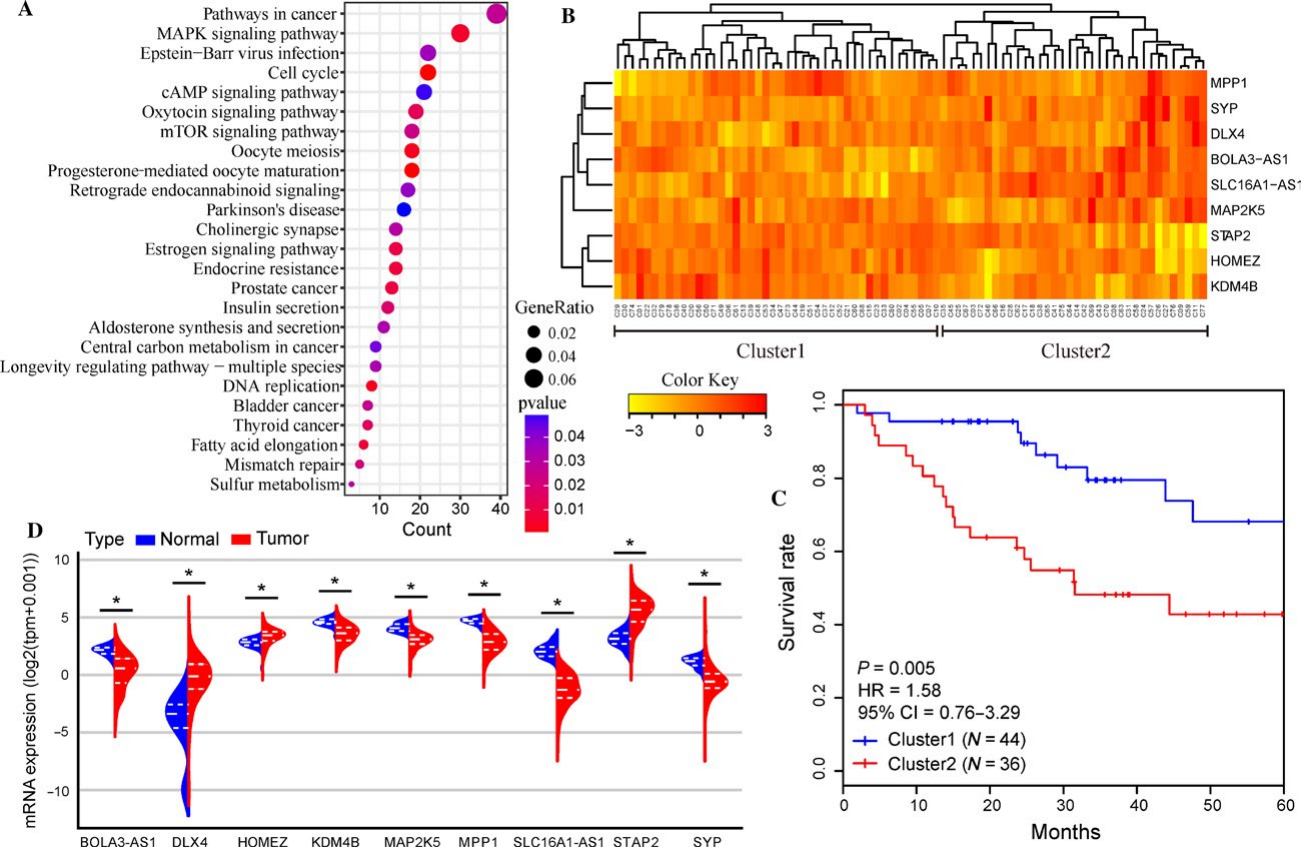
Identification of optimal signature genes for overall survival (OS) prediction in endometrial carcinoma (EC). (A) Functional enrichment analysis of Kyoto Encyclopedia of Genes and Genomes for 1571 genes associated with OS. Only pathways with a *P*‐value <0.05 are presented. (B) The unsupervised hierarchical clustering heatmap of the training dataset based on the expression profiles of nine signature genes. Patients were categorized into two clusters. (C) Kaplan–Meier curves for patients in two different clusters. (D) The mRNA expression of nine signature genes in 181 EC tissues and 78 normal uterine tissues in the dataset from the UCSC Xena project. The distribution of expression data is represented by a violin plot, and the dashed lines indicate the quartiles. *P*‐values were calculated by Mann–Whitney *U* test. (*P < 0.001)

To further understand the molecular function and pathway of these genes associated with survival, these 1571 genes were included in functional enrichment analysis of KEGG pathways. The hypergeometric test statistical method was employed, and the pathways with a *P*‐value threshold of <0.05 were considered as significant enriched pathways. As a result, these genes were enriched in total 25 pathways (Fig. 1A). In detail, the genes are associated with various signaling pathways, such as the mitogen‐activated protein kinase (MAPK) signaling pathway, estrogen signaling pathway, oxytocin signaling pathway and mTOR signaling pathway, and several pathways in cancer, including thyroid cancer, bladder cancer, and prostate cancer (Fig. 1A).

### Selection of optimal signature genes for OS prediction

These 1571 genes were further narrowed down to select optimal signature genes for prognosis prediction. A series of gene combinations were generated using stepwise selection, and the optimal combination was finally selected based on the minimum Akaike information criterion. Signature genes, including *SLC16A1‐AS1*,* KDM4B*,* MAP2K5*,* SYP*,* MPP1*,* DLX4*,* BOLA3‐AS1*,* HOMEZ,* and *STAP2*, were chosen to optimally predict the OS of EC patients. The selected genes were next subjected to unsupervised hierarchical clustering analysis, and the EC patients were then divided into two groups: Cluster1 and Cluster2 (Fig. 1B). A significant difference was observed between the OS of these two groups by Kaplan–Meier curves (*P* = 0.005, Fig. 1C), indicating that these nine signature genes might be used to predict the OS of EC patients.

### Differential expression of nine signature genes between EC and normal uterine tissues

The mRNA expressions of these nine signature genes between EC and normal uterine tissues were compared using 259 samples (181 EC tissues and 78 normal uterine tissues) from the UCSC Xena project. The mRNA expression of *SLC16A1‐AS1*,* KDM4B*,* MAP2K5*,* SYP*,* MPP1,* and *BOLA3‐AS1* was significantly down‐regulated in 181 EC samples compared with 78 normal uterine tissues (all *P* < 0.001, Fig. 1D). On the other hand, *DLX4*,* HOMEZ,* and *STAP2* were overexpressed in EC tissues (all *P* < 0.001, Fig. 1D). These findings suggested that these nine signature genes may be involved in the development of EC.

### Construction of a prognostic model based on nine signature genes

**Figure 2:**
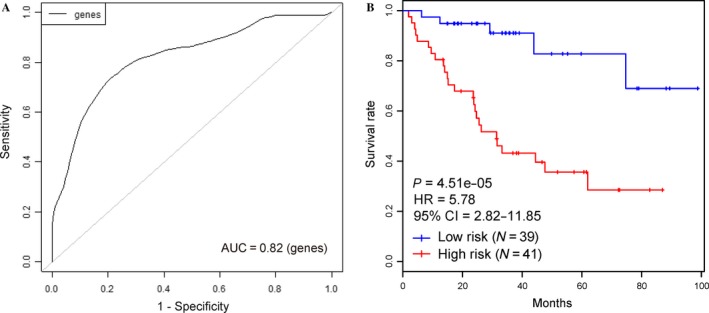
Construction of prognostic model based on nine signature genes. (A) The cross‐validated time‐dependent ROC curve for survival predictions was produced using BRB‐ArrayTools. (B) Kaplan–Meier curves for patients in two risk groups that were partitioned based on the 50th prognostic index percentile.

The expression level of the nine signature genes in eighty training samples was used to construct the survival risk score system (prognostic model) with the penalized Cox regression method, and the regression coefficient for each gene was subsequently obtained (Table 1). In detail, the survival risk score can be calculated based on the following formula: risk score = (0.21 × expression level of *SLC16A1‐AS1*) + (−0.877 × expression level of *KDM4B*) + (0.852 × expression level of *MAP2K5*) + (−0.046 × expression level of *SYP*) + (0.482 × expression level of *MPP1*) + (0.155 × expression level of *DLX4*) + (0.4 × expression level of *BOLA3‐AS1*) + (−0.384 × expression level of *HOMEZ*) + (0.012 × expression level of *STAP2*). From the formula above, a higher score indicates an increased risk of mortality, whereas a lower score denotes a better outcome. LOOCV was performed to evaluate the prediction accuracy of this model, and the cross‐validated time‐dependent ROC curve was plotted. The area under the ROC curve (AUC value) was 0.82 (Fig. 2A), verifying the ability of this model for OS prediction. The eighty EC patients were further partitioned into two risk groups based on the 50th prognostic index percentile. The Kaplan–Meier curve for these two risk groups revealed that the OS for patients in the high‐risk group was significantly poorer than that in low‐risk group (HR = 5.78, *P* < 0.001, Fig. 2B).

**Table 1:** Nine signature genes included in the prognostic model

Coefficient	Cross‐validation support (%)	EntrezID	Symbol	Gene expression level association with poor prognosis
0.21	100	100506392	SLC16A1‐AS1	High
−0.877	100	23030	KDM4B	Low
0.852	100	5607	MAP2K5	High
−0.046	98.75	6855	SYP	Low
0.482	100	4354	MPP1	High
0.155	100	1748	DLX4	High
0.4	100	100507171	BOLA3‐AS1	High
−0.384	100	57594	HOMEZ	Low
0.012	90	55620	STAP2	High

### Performance evaluation of the nine‐gene prognostic model

**Figure 3:**
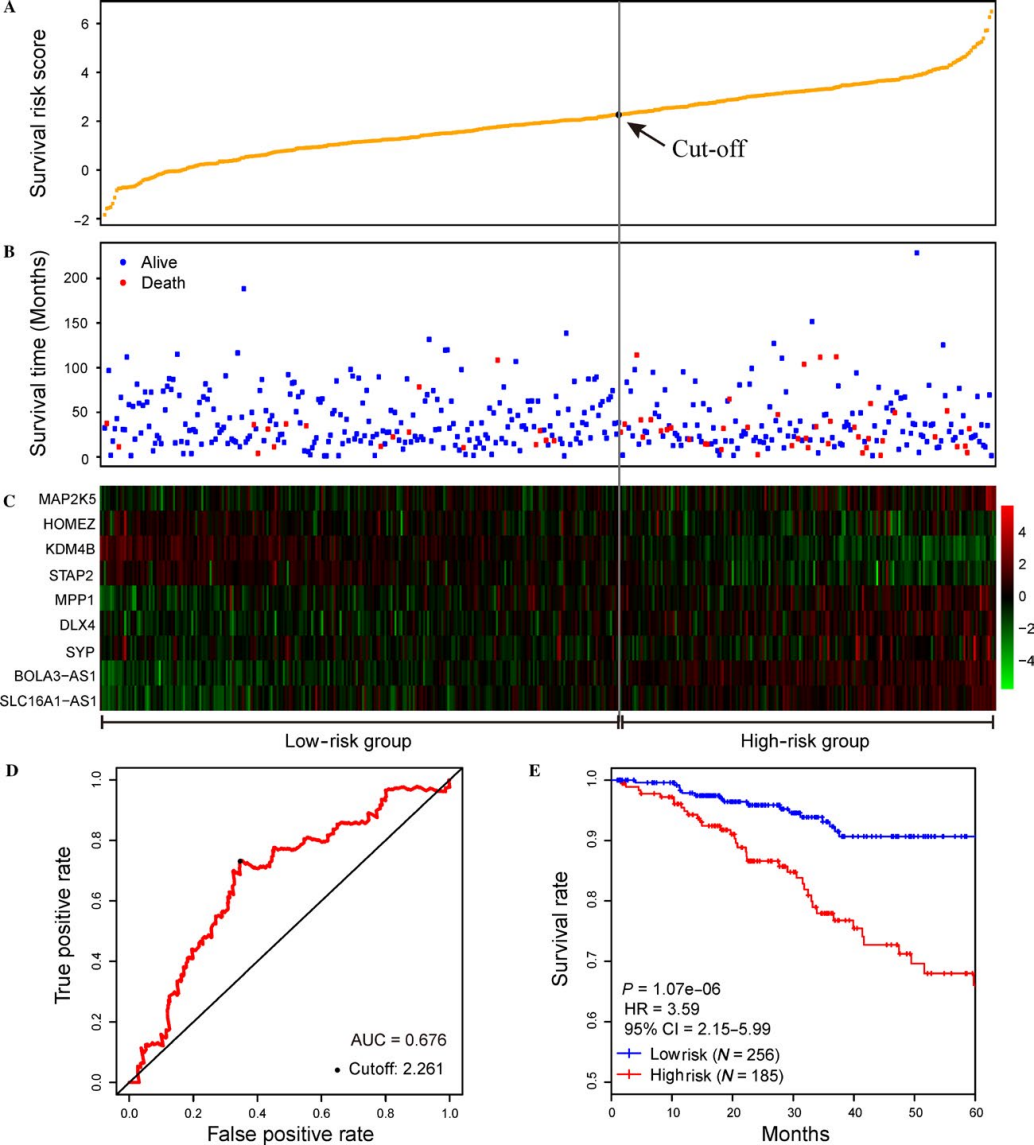
Performance of the nine‐gene model in overall survival (OS) prediction of endometrial carcinoma (EC) using the validation dataset. (A) The distribution of survival risk score of EC patients in validation dataset. (B) Survival (or censoring) time of EC patients. (C) Clustering heatmap of mRNA expression profiles of the nine signature genes. (D) The ROC curve was generated for 5‐year OS predictions with an AUC of 0.676. The optimal cutoff value (2.261), shown as the gray straight line in A, B, and C, was obtained to divide the patients into low‐ and high‐risk groups. (E) Kaplan–Meier curves for patients in two risk groups. Patients in the high‐risk group exhibited a poorer OS compared with patients in the low‐risk group (HR = 3.589, *P* < 0.001).

The validation dataset, including 441 EC samples (Fig. 3B), was used to evaluate the robustness and effectiveness of the nine‐gene prognostic model. Based on expression level of the nine signature genes in the validation dataset, the survival risk scores were calculated for each patient (Fig. 3A). ROC curve analysis for 5‐year OS prediction was performed with an AUC of 0.676 (Fig. 3D), confirming the ability of this nine‐gene model to predict prognosis in EC patients. The patients were divided into two risk groups (Fig. 3B and C) based on the optimal cutoff risk score (2.261, Fig. 3D) that was determined by the maximum Youden index in the ROC curve. In detail, 185 (41.95%) patients were classified as the high‐risk group, whereas the remaining 256 (58.05%) patients were categorized as the low‐risk group. A significant difference between the 5‐year OS of the 2 risk groups was demonstrated by Kaplan–Meier curve analysis (HR = 3.59, *P* < 0.001, Fig. 3E). The analogous situation was noted for the entire TCGA dataset (AUC = 0.713, HR = 4.18, *P* < 0.001, Fig. S1A and B). A panel of nine signature genes proposed by O'Mara et al. 20 was compared with our model using the entire TCGA dataset, which indicated an AUC of 0.685 in ROC curve (Fig. S1C) and a significant difference between high‐ and low‐risk group (Fig. S1D).

### Comparison of OS prediction power of the nine‐gene prognostic model with the FIGO staging and histological typing

**Figure 4:**
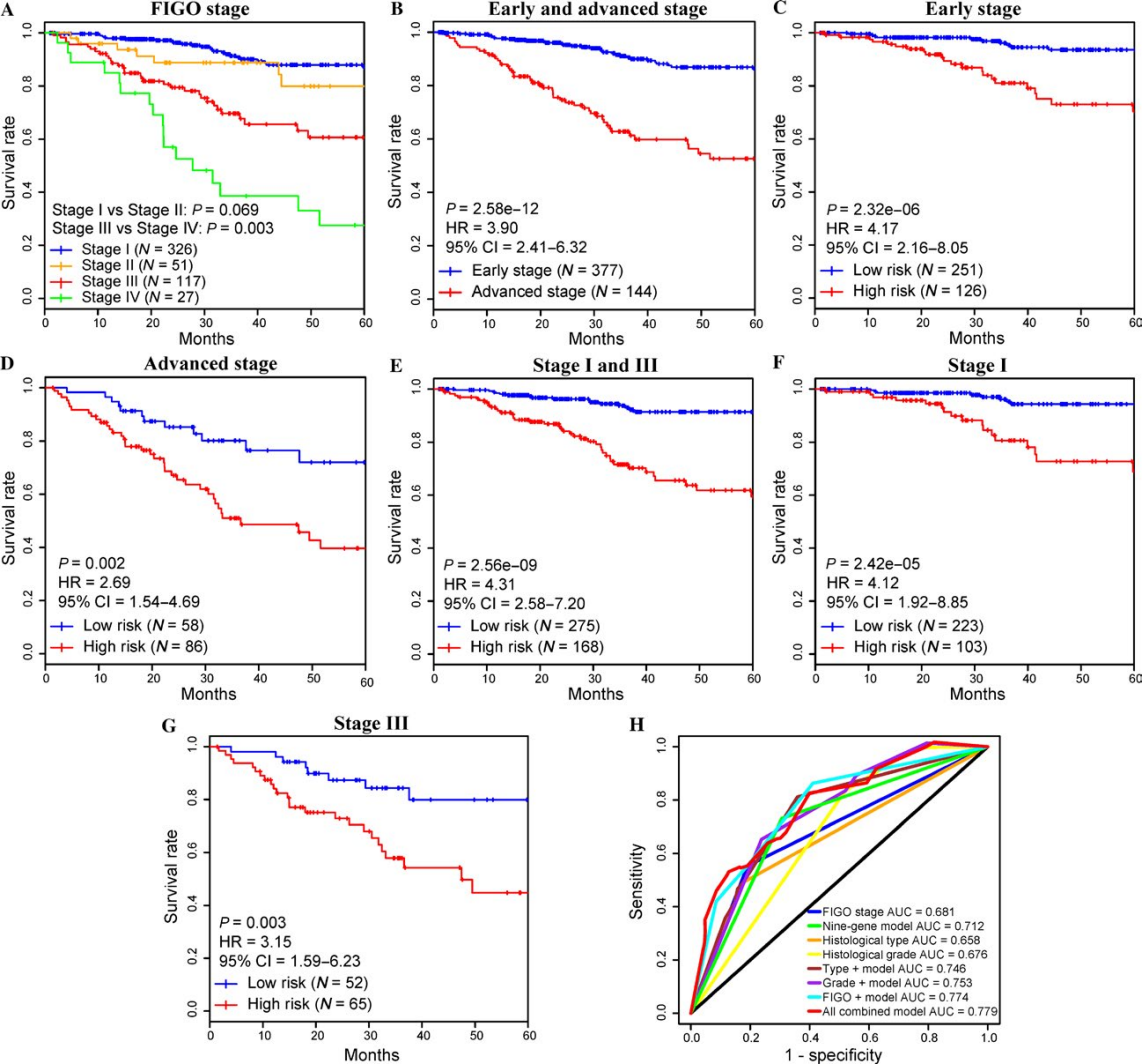
Comparison of survival prediction power of the nine‐gene prognostic model with the FIGO stage. (A) Kaplan–Meier curves for patients in four FIGO stages. A significant difference was observed between 5‐year overall survival (OS) of stage III and stage IV (in advanced stage) patients (*P* = 0.003), whereas no significant difference was noted between stage I and stage II (in early stage) (*P* > 0.05). (B) For 521 endometrial carcinoma (EC) patients, advanced stage EC was associated with increased 5‐year mortality compared with early stage EC. (C, D, E, F, G) The EC patients in various stages were divided into high‐ and low‐risk groups based on their survival risk scores. By plotting Kaplan–Meier curves, the nine‐gene model for prediction of 5‐year OS in patients with early stage (C) and advanced stage (D) EC was assessed individually. Similarly, the association between the prognostic model (survival risk) and 5‐year OS in patients with stage I and stage III EC was also evaluated simultaneously (E) or individually (stage I in (F); stage III in (G)). (H) The ROC curves for OS prediction of the FIGO stage, histological type, histological grade, the nine‐gene prognostic model, and the combined model.

To include more EC samples in this section, the entire TCGA dataset (*N* = 521) was used to evaluate this nine‐gene model with respect to the prognosis among patients based on FIGO stage. Kaplan–Meier curves using the TCGA dataset based on FIGO stage (Fig. 4A) and early (stage I and II)/advanced (stage III and IV) stage (Fig. 4B) were plotted to depict the relationship between FIGO stage and OS prediction, respectively. A significant difference was observed between stage III and stage IV (*P* = 0.003, Fig. 4A) in advanced stage, whereas no obvious association was observed between stage I and stage II (*P* > 0.05, Fig. 4A) in early stage. Additionally, advanced stage cancers were associated with increased 5‐year mortality compared with early stage cancers (HR = 3.90, *P* < 0.001, Fig. 4B). To assess the effectiveness of this nine‐gene model among patients in different FIGO stages, the association between risk scores and OS prediction was also investigated. Compared with the low‐risk group, the high‐risk group exhibited significantly increased five‐year mortality for early stage (HR = 4.17, *P* < 0.001, Fig. 4C) and advanced stage EC (HR = 2.69, *P* = 0.002, Fig. 4D). The performance of nine‐gene model for patients with FIGO stage I and stage III was also assessed given the relatively large sample of stage I and stage III patients in the TCGA dataset. Similarly, patients in the high‐risk group exhibited reduced survival rate compared with the low‐risk group (HR = 4.31, *P* < 0.001, Fig. 4E). In addition, the high‐risk group in stage I (HR = 4.12, *P* < 0.001, Fig. 4F) and stage III (HR = 3.15, *P* = 0.003, Fig. 4G) also exhibited significantly increased 5‐year mortality rates. Therefore, this nine‐gene model might be used to predict prognosis in EC patients both with early and advanced stages.

Performance of this model was also evaluated based on the three separated histological types of EC. A significant difference was observed between 5‐year OS of EEA (endometrioid endometrial adenocarcinoma) and MSE (mixed serous and endometrioid, *P* < 0.001), and EEA and SEA (serous endometrial adenocarcinoma, *P* = 0.013) patients, whereas no significant difference was noted between MSE and SEA (*P* > 0.05, Fig. S2A). Based on this model, Kaplan–Meier analysis for OS demonstrated a significant difference between the groups predicted to be high risk or low risk both in EEA (*P* < 0.001, Fig. S2B) and MSE (*P* = 0.026, Fig. S2C). However, no obvious difference was observed between two risk groups in SEA (*P* > 0.05, Fig. S2D), which might have been due to the relatively elevated risk of mortality in SEA (90/110) compared with the other two types (112/390 for EEA and 10/21 for MSE) of EC.

Several potential prognostic factors, including age, FIGO staging system, histological grade, histological type, and survival risk (nine‐gene prognostic model), were included in univariate (*P* < 0.001, Table 2) and multivariate‐adjusted Cox regression analyses, indicating the relatively high prognostic significance of this nine‐gene model for 5‐year OS risk in the TCGA dataset (*N* = 521, *P* = 0.001, Table 3). These findings suggested that this model might be an independent classifier for the prognostic prediction of EC patients. Subsequently, ROC curve analysis was performed to compare the sensitivity and specificity in OS prediction among some of these prognostic factors (FIGO staging system, histological grade, histological type, and this prognostic model, Fig. 4H). Here, we assumed that the larger area under the ROC curve (AUC) usually implies a better model for prediction 22. In the entire TCGA dataset (*N* = 521), the predictive ability of the nine‐gene prognostic model was significantly better than the FIGO stage (AUC = 0.712) and other single factors (Fig. 4H), further demonstrating that the model in our study is a novel prognostic marker with higher accuracy and has important clinical significance. Remarkably, the combined models especially for our model combined with FIGO stage (AUC = 0.774) or all other factors (AUC = 0.779, Fig. 4H) had larger AUC values than the FIGO stage, histological type, histological grade, or our model alone (Fig. 4H), suggesting that this nine‐gene model might be used to assist prognosis prediction for EC patients combined with clinical factors such as FIGO stage.

**Table 2:** Univariate Cox regression analysis of potential prognostic factors for endometrial carcinoma patients in the TCGA dataset

Characteristics	No. of patients	5‐year SR (%)	*P*‐value
Age			
≤60	171	85.4	0.002
>60	350	71.8	
FIGO stage			
I	326	86.9	4.02E‐11
II	51	76.5	
III	117	60.7	
IV	27	29.4	
Histological grade			
G1	95	97.2	1.90E‐08
G2	116	81.7	
G3	310	67.7	
Histological type			
EEA	390	82.5	8.71E‐06
MSE	21	57.8	
SEA	110	57.6	
Survival risk			
Low	309	88.2	4.31E‐11
High	212	59.0	

EEA, Endometrioid endometrial adenocarcinoma; MSE, Mixed serous and endometrioid; SEA, Serous endometrial adenocarcinoma.

**Table 3:** Multivariate‐adjusted Cox regression analysis of potential prognostic factors for endometrial carcinoma patients in the TCGA dataset

Characteristics	Adjusted HR	95% CI for HR		*P*‐value
		Lower	Upper	
Age				
≤60	1 (ref)			
>60	1.503	0.879	2.571	0.136
FIGO stage				
I	1 (ref)			
II	1.354	0.639	2.868	0.429
III	2.488	1.490	4.154	4.90e‐04
IV	4.849	2.585	9.096	8.74e‐07
Histological grade				
G1	1 (ref)			
G2	6.030	1.363	26.684	0.018
G3	5.568	1.310	23.667	0.020
Histological type				
EEA	1 (ref)			
MSE	1.998	0.825	4.840	0.125
SEA	1.065	0.644	1.761	0.807
Survival risk				
Low	1 (ref)			
High	2.413	1.421	4.097	0.001

EEA, Endometrioid endometrial adenocarcinoma; MSE, Mixed serous and endometrioid; SEA, Serous endometrial adenocarcinoma.

Adjusted factor is age.

## Discussion

In this study, eighty samples were included in training dataset, and a total of 1571 genes were identified as OS‐related genes based on genomewide expression profiles. Among them, partial genes have been previously reported to be associated with EC prognosis, such as *TP53* 23, 24, *PIK3CA* 25, *CDKN2A* 26, 27, and *PTEN* 17. By performing KEGG enrichment analysis (Fig. 1A), these OS‐related genes were enriched in several pathways in cancers, including thyroid cancer, bladder cancer, and prostate cancer. Moreover, significant enrichment of these genes in various signaling pathways, such as the MAPK signaling pathway, estrogen signaling pathway, oxytocin signaling pathway, and mTOR signaling pathway, was also observed. Estrogen regulates various physiological responses in numerous target tissues and plays important roles in the development and progression of breast cancers 28, making it a therapeutic target for cancer therapy 29. Oxytocin may play a regulatory role in tumor growth 30, and the presence of the oxytocin receptor in endometrial cancer cells represents a key factor in endometrial cancer progression 31. Hyperactivation of the mTOR pathway increases cell growth and proliferation and stimulates tumor growth, representing a potential therapeutic target of cancers. The Ras‐activated MAPK signaling pathway has been well studied 32 and regulates the transcription of genes that are important in the cell cycle 33. The optimal signature genes were further chosen by stepwise selection and finally included *SLC16A1‐AS1*,* KDM4B*,* MAP2K5*,* SYP*,* MPP1*,* DLX4*,* BOLA3‐AS1*,* HOMEZ,* and *STAP2* (Table 1). These genes were subsequently used to construct a prognostic model for OS prediction of EC. As for the characteristics of these signature genes, higher expression levels of *SLC16A1‐AS1*,* MAP2K5*,* MPP1*,* DLX4*,* BOLA3‐AS1,* and *STAP2* are associated with poor prognosis. On the other hand, higher expression levels of the remaining *KDM4B*,* SYP,* and *HOMEZ* are associated with longer OS (Table 1). It is noteworthy that some of these genes have been reported in previous studies of cancer. *KDM4B* represents a novel prognostic factor for resected lung adenocarcinoma 34 and a potential diagnostic marker for human hepatocellular carcinoma 35. Several studies have demonstrated that MAP2K5 plays an important role in the development of prostate cancer 36, breast cancer 37, and hepatocellular carcinoma 38. High expression of *DLX4* is strongly associated with survival of ovarian cancer patients 39. *HOMEZ*,* MPP1*,* STAP2,* and *SYP* are potentially involved in cellular metabolism and regulation. Additionally, two noncoding RNA genes (*SLC16A1‐AS1* and *BOLA3‐AS1*) that are the antisense RNAs of *BOLA3* and *SLC16A1* were also included in this model. However, their functions remain unknown based on searches using NCBI (http://www.ncbi.nlm.nih.gov/gene/) and GeneCards (http://www.genecards.org/). Interestingly, several long noncoding RNAs also have been identified as biomarkers associated with EC progression and patient outcome in a recent study 7. Remarkably, these nine signature genes were differentially expressed between EC and normal uterine tissues (Fig. 1D) by mRNA expression analysis, indicating their potential role in the development of EC. Briefly, these nine genes not only were associated with OS for EC patients but also might represent novel oncogenes or tumor suppressor genes that require further study.

The prognostic model was ultimately constructed based on these nine signature genes. Subsequently, cross‐validated time‐dependent ROC curves (Fig. 2A) and Kaplan–Meier plots (Fig. 2B) were both employed to evaluate the prediction accuracy of this model. Further validation procedure was conducted using the validation dataset and entire TCGA dataset, and ROC curve analysis confirmed the robustness and effectiveness of this model to predict OS in EC patients with AUC values of 0.676 and 0.713, respectively (Fig. 3D and Fig. S1A).

The FIGO staging system and the histological typing are the most‐adopted classification for the treatment and prognosis for EC patients 4, 40. Indeed, the discriminating power of FIGO stage was observed for advanced stage (Fig. 4A), but not for early stage EC, which is consistent with a previous study 12. A similar case arose when evaluated based on the separated histological types of EC (Fig. S2A). These findings demonstrated the limitation of the FIGO staging system and histological typing to accurately predict the prognosis of EC. Based on the TCGA dataset, this nine‐gene model also exhibited the ability to predict the prognosis for patients not only with FIGO staging (early and/or advanced stage, Fig. S1B, Fig. 4C and D; stage I and/or stage III, Fig. 4E–G) but with histological types (EEA and MSE, Fig. S2B and C) of EC.

Molecular classification of EC has been shown to be reproducible and associated with clinical outcomes 41, 42. An integrated genomic‐pathologic classification of EC has been proposed 10, 41, which defined four major classes of tumor (POLE‐ultramutated, microsatellite instability–hypermutated [MSI‐H], copy‐number‐low, and copy‐number‐high). Remarkably, a significant association was observed between our model and this molecular classification (*P* < 0.001, Table S3), which confirmed the predictive ability of this model and suggested that this model may also be an alternative or complementary method for molecular classification of EC.

Remarkably, this model was also demonstrated to be an independent prognostic factor for predicting OS of EC patients based on univariate and multivariate analysis (Tables 2 and 3). Additionally, this model can further distinguish patients with an elevated risk of mortality based on the FIGO staging system and has a more powerful ability for prognosis prediction combined with FIGO stage and/or other histological classifications (Fig. 4H). Therefore, our model may be used to assist the FIGO stage to predict EC patient prognosis, contributing to rational therapy and avoiding inadequate or excessive treatment. Compared with other panels of biomarkers constructed in other studies 20, our model achieved a similar or slightly better effect (Fig. S1). Additionally, this model was established based on genomewide gene expression profiles, including all protein‐coding and RNA genes produced by RNA‐Seq and contains only a few signature genes that could effectively predict the OS of EC patients. This model will be efficiently applied to clinical practice once the RT‐PCR assay including these nine signature genes is developed. However, due to the limited sample size, more EC samples are required to further prove the prognostic value of this model in EC patients before it is applied in clinically.

In general, we present and validate a robust prognostic model aggregating nine signature genes based on genomewide expression profiles that can be used to efficiently predict EC patient prognosis. Using this model, we could further distinguish patients with an elevated risk of mortality based on the FIGO staging system and other prognostic factors, which may help to guide the application of rational therapy in clinical practice. In addition, this study will help to improve our understanding of underlying mechanisms involved in EC prognostic factors.

## Conflict of Interest

The authors declare no conflict of interests.

## Supporting information


**Figure S1.** Comparison of the nine‐gene prognostic model with other prognostic classifiers.

**Figure S2.** Performance of the nine‐gene model in OS prediction of EC based on the three separated histological types.

**Table S1.** Characteristics of EC samples used in this study.

**Table S2.** 1571 genes associated with OS identified in EC.

**Table S3.** Association of the nine‐gene prognostic model with molecular classification in EC patients (*n* = 230).
