# Supplementary material for: Establishment of a nine‐gene prognostic model for predicting overall survival of patients with endometrial carcinoma
Source: Cancer Med. 2018 Apr 17;7(6):2601–11. doi: 10.1002/cam4.1498 (PMC6010780; doi:10.1002/cam4.1498)
Supplement: Supplementary file 1 — Figure S1. Comparison of the nine‐gene prognostic model with other prognostic classifiers. [file CAM4-7-2601-s001.docx]

**Supplementary Figure**


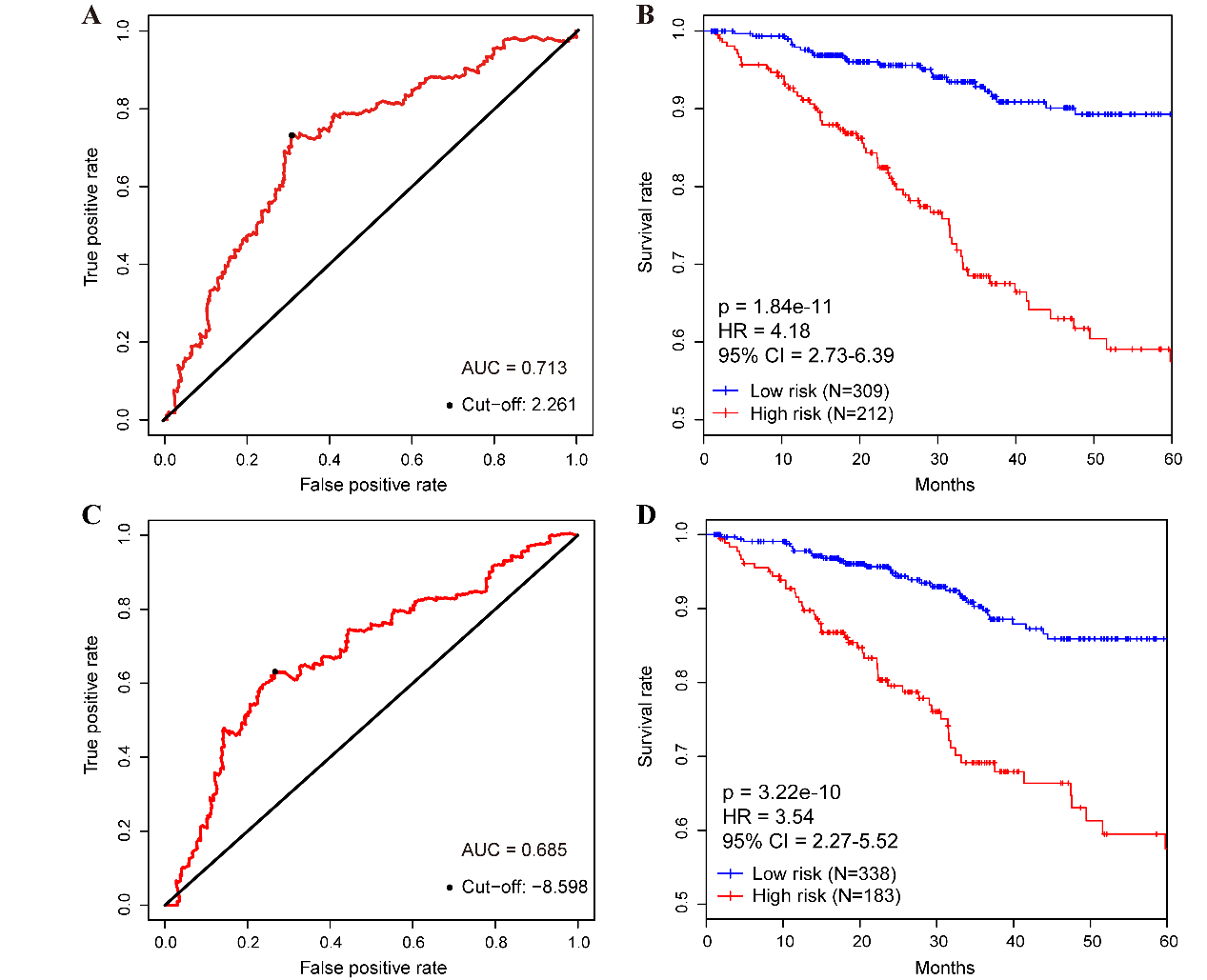


Supplementary Figure S1. Comparison of the nine-gene prognostic model with other prognostic classifiers. (A, B) Performance of the nine-gene model in OS prediction of EC using the entire TCGA dataset (N = 521). (A) The ROC curve was generated for 5-year OS predictions with an AUC of 0.713. The optimal cut-off value (2.261) was obtained to divide the patients into low- and high-risk groups. (B) Kaplan-Meier curves for patients in 2 risk groups. Patients in the high-risk group had poorer OS compared with patients in the low-risk group (HR = 4.18, p < 0.001). (C, D) Performance of the nine-gene signature (*PDLIM1*, *FBP1*, *NLRC3*, *ST6GALNAC1*, *C4BPA*, *PPP2R3A*, *TRIM46*, *EPH2* and *PRRG1*) proposed by O'Mara *et al.* in OS prediction of EC using the entire TCGA dataset (n = 521). (C) The ROC curve was generated for 5-year OS predictions with an AUC of 0.685. The optimal cut-off value (-8.598) was obtained to divide the patients into low- and high-risk groups. (D) Kaplan-Meier curves for patients in 2 risk groups. Patients in the high-risk group had poorer OS compared with patients in the low-risk group (HR = 3.54, p < 0.001).
