# Supplementary material for: Establishment of a nine‐gene prognostic model for predicting overall survival of patients with endometrial carcinoma
Source: Cancer Med. 2018 Apr 17;7(6):2601–11. doi: 10.1002/cam4.1498 (PMC6010780; doi:10.1002/cam4.1498)
Supplement: Supplementary file 2 — Figure S2. Performance of the nine‐gene model in OS prediction of EC based on the three separated histological types. [file CAM4-7-2601-s002.docx]

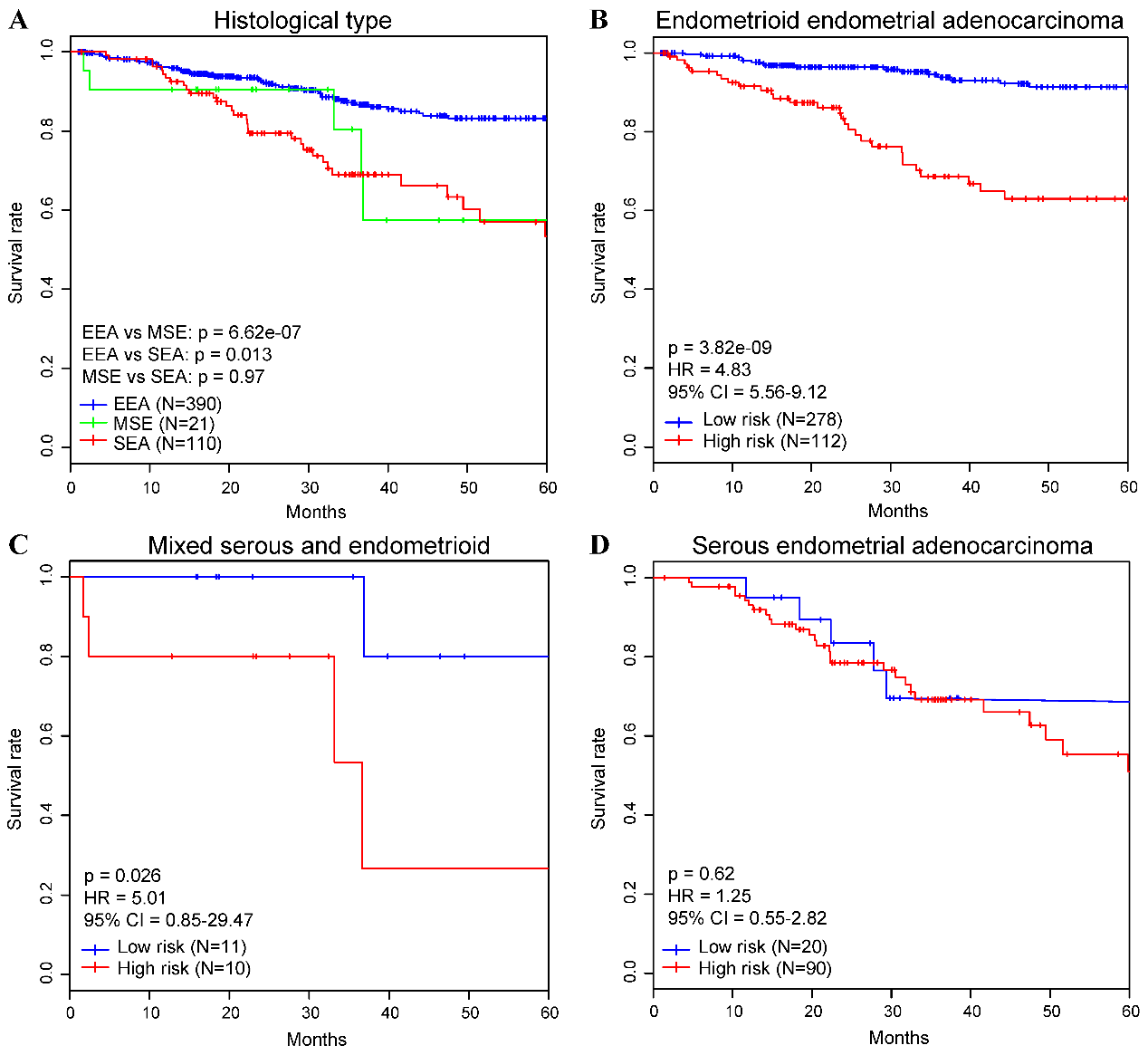


Supplementary Figure S2. Performance of the nine-gene model in OS prediction of EC based on the three separated histological types. (A) Kaplan-Meier curves for patients in three histological types. A significant difference was observed between 5-year OS of EEA (endometrioid endometrial adenocarcinoma) and MSE (mixed serous and endometrioid) (p < 0.001), and EEA and SEA (serous endometrial adenocarcinoma) patients (p = 0.013), whereas no significant difference was noted between MSE and SEA (p > 0.05). (B, C, D) The EC patients in various histological types were divided into high- and low-risk groups based on their survival risk scores. By plotting Kaplan-Meier curves, the nine-gene model for prediction of 5-year OS in patients with EEA (B), MSE (C) and SEA (D) was assessed individually.
